# Risk Factors for Severe Hand-Foot-Mouth Disease in China: A Systematic Review and Meta-Analysis

**DOI:** 10.3389/fped.2021.716039

**Published:** 2021-11-10

**Authors:** Peiqing Li, Yuge Huang, Danping Zhu, Sida Yang, Dandan Hu

**Affiliations:** ^1^Department of Pediatric Emergency, Guangzhou Women and Children's Medical Center, Guangzhou Medical University, Guangzhou, China; ^2^Pediatric Intensive Care Unit, Affiliated Hospital of Guangdong Medical University, Zhanjiang, China; ^3^Department of Pediatric Neurology, Guangzhou Women and Children's Medical Center, Guangzhou Medical University, Guangzhou, China; ^4^Children's Health Section, Guangzhou Women and Children's Medical Center, Guangzhou Medical University, Guangzhou, China

**Keywords:** hand-foot-mouth disease, infection, meta-analysis, risk factors, disease severity

## Abstract

**Background:** This study aimed to identify potential risk factors for severe hand-foot-mouth disease (HFMD).

**Methods:** The PubMed, Embase, the Cochrane Library, Sinomed, WanFang, CNKI, and VIP databases were searched (up to August 2021).

**Results:** Twenty-nine studies (9,241 and 927,355 patients with severe HFMD and controls, respectively; all from China) were included. EV71 was associated with higher odds of severe HFMD compared with other agents (OR = 4.44, 95%CI: 3.12–6.33, *p* < 0.001). Being home-raised (OR = 1.99, 95%CI: 1.59–2.50, *p* < 0.001), higher number of children in the family (OR = 2.09, 95%CI: 1.93–2.27, *p* < 0.001), poor hand hygiene (OR = 2.74, 95%CI: 1.78–4.23, *p* < 0.001), and no breastfeeding (OR = 2.01, 95%CI: 1.45–2.79, *p* < 0.001) were risk factors for severe HFMD. First consulting to a district-level or above hospital (OR = 0.34, 95%CI: 0.25–0.45, *p* < 0.001) and diagnosis of HFMD at baseline (OR = 0.17, 95%CI: 0.13–0.24, *p* < 0.001) were protective factors against severe HFMD. Fever, long fever duration, vomiting, lethargy, leukocytosis, tic, and convulsions were each associated with severe HFMD (all *p* < 0.05), while rash was not.

**Conclusions:** EV71, lifestyle habits, frequent hospital visits, and symptoms are risk factors for severe HFMD in children in China, while early diagnosis and admission to higher-level hospitals are protective factors.

## Introduction

Hand-foot-mouth disease (HFMD) is widespread among children and is generally characterized by fever, oral ulcers, and rash on the hands, feet, and buttocks (1–3). The main causative pathogens include human enterovirus 71 (EV 71), Coxsackievirus A16 (CA 16), and other enteroviruses (1–3). The common route of transmission of HFMD is through the fecal-oral route, respiratory droplets, contact with blister fluid, or close contact with infected children (1, 2). Although most cases are mild and self-limited, HFMD is a serious public health concern in children throughout Asia (4). Indeed, the majority of outbreaks are reported in Malaysia, Vietnam, China, Cambodia, and India (2), with an incidence of 115–197 cases per 100,000 person-years (5, 6). Severe or fatal HFMD cases have been reported in epidemiological studies as being frequent in Malaysia, Japan, Vietnam, and China and have received extensive attention from the global public health sector (4, 7–10). Studies from China have reported 27,908 children with HFMD and 905 children with fatal HFMD in 2010 (11, 12).

The most common manifestations of fatal HFMD are encephalitis and subsequent pulmonary edema, but myocarditis, neurologic complications, and paroxysmal tachycardia have been reported (1–3, 8, 13–15). Moreover, brainstem and thalamus symptoms occur at an early stage and are associated with a poor prognosis of HFMD (16).

Therefore, it is important to examine the biological and behavioral characteristics of children to identify the risk factors associated with progression to severe HFMD due to the additional fatality risk in severe cases. Several factors were shown to be associated with an increased risk of severe HFMD in China (17). Various risk factors for severe HFMD were reported in the literature (18–20), but there is a lack of consensus because of different factors being analyzed in the first place, different outcomes, and different study populations.

Hence, these results were not consistent, so continued investigations are needed to confirm known and identify additional potential risk factors. The current meta-analysis was conducted to explore the potential factors associated with the progression to severe HFMD.

## Methods

### Data Sources, Search Strategy, and Selection Criteria

This study was conducted and reported according to the Preferred Reporting Items for Systematic Reviews and Meta-Analysis (PRISMA) Statement (21, 22). Any study (irrespective of the language) that investigated the risk factors for severe HFMD was eligible for inclusion in this study. The electronic databases of PubMed, Embase, the Cochrane Library, Sinomed, WanFang, CNKI, and VIP were systematically searched from their inception up to August 2021. The core search terms included “hand foot mouth disease” OR “coxsackievirus a16” OR “enterovirus 71” AND “risk factors.” The reference lists from the retrieved studies were also reviewed to identify any potentially eligible studies.

Two authors independently conducted the literature search and study selection following a standardized form, and any disagreement was settled by a third author. The inclusion criteria were (1) study design: prospective or retrospective observational study, (2) participants: individuals with HFMD, (3) exposure: the study reported the effect estimates of potential risk factors for severe HFMD, (4) outcomes: severe HFMD [i.e., the development of nervous system involvement, physical signs of meningeal irritation, or weakened or missing tendon reflexes (23)]; and (5) sample size >30. Severe HFMD was diagnosed according to the Chinese guidelines that were current when the included studies were conducted; the most recent version is 2018 (24).

### Data Collection and Quality Assessment

The data abstraction and quality assessment were conducted by two authors, and any inconsistency was resolved by a third author after referring to the original article. The following variables were collected: first author's surname, publication year, study design, country, sample size, number of boys and girls, mean age, the definition of severe cases, and reported factors. The study quality was assessed using the Newcastle-Ottawa score (NOS) scale, which is based on selection (four items), comparability (one item), and outcome (three items), and the star system ranges from 0 to 9 (25).

### Statistical Analysis

The odds ratios (ORs) and their 95% confidence intervals (CIs) were extracted from the multivariable logistic regressions presented in the original papers. The summary ORs were calculated using the random-effects model to account for heterogeneity in study design and populations among studies (26, 27). Heterogeneity across included studies was assessed by *I*^2^- and *Q*-statistics, and *p*-value < 0.10 was regarded as significant heterogeneity (28, 29). Sensitivity analyses were conducted for risk factors reported in more than four studies to assess the impact of a single study on the overall analysis (30), as well as for EV 71. Publication biases were evaluated using the funnel plot, Egger test (31), Begg test (32), and trim and fill analysis (33). Two-sided *p* < 0.05 was considered statistically significant. All statistical analyses were conducted using STATA 10.0 (Stata Corporation, College Station, TX, USA).

## Results

### Literature Search

**Figure 1:**
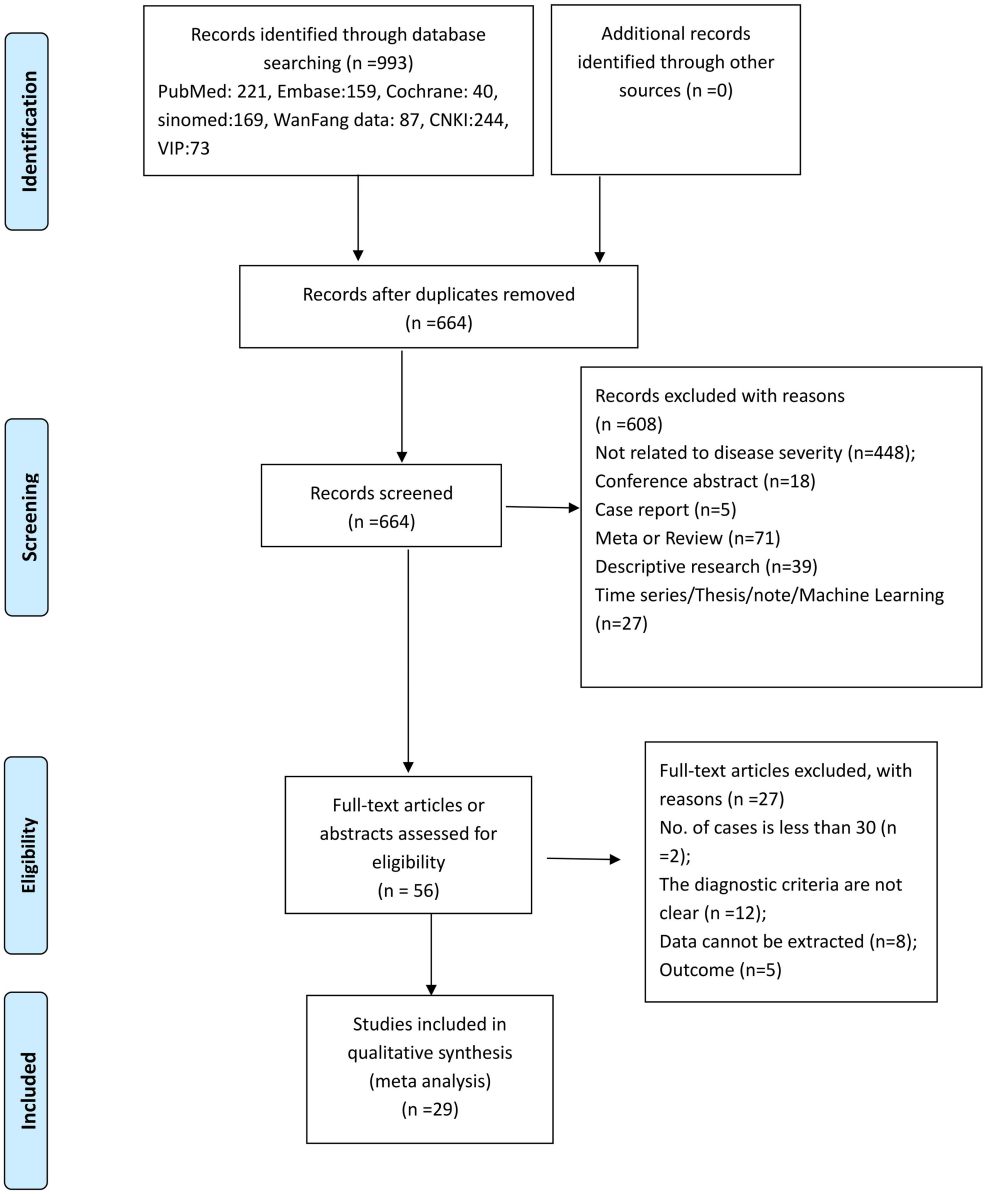
PRISMA 2009 flow diagram of the study selection process.

Figure 1 presents the study selection process. The initial search yielded 993 entries, and 664 were screened after removing the duplicates. From these, 608 were excluded, and 56 full-text papers were screened. Then, 27 studies were excluded (sample size, *n* = 2; unclear diagnostic criteria, *n* = 12; data could be extracted, *n* = 8; outcome, *n* = 5). Finally, 29 studies were included.

### Characteristics of the Studies

Table 1 presents the characteristics of the studies. There were 19 case-control studies, seven matched case-control studies, and two nested case-control studies. There were 9,241 patients with severe HFMD (31–3,738/study) and 927,355 controls (37–920,011/study). Regarding the NOS, nine studies scored five stars, seven scored six stars, eleven scored seven stars, and two scored eight stars out of a possible total of nine stars.

**Table 1:** Characteristics of the included studies.

	**Country and province**	**Study design**	**Sample size (cases/controls)**	**Mean age (cases/controls, month)**	**Sex [cases/controls, males (%)]**	**Guidelines version for the diagnosis and treatment**	**Total quality score (NOS)**
Li and Xu (34)	China, Shenyang	Case-control	112/224	NA	57.1/60.3	2018	7
Liu and Qi (35)	China, Shenzhen	Case-control	131/174	20.6/20.5	64.1/55.8	2018	7
Wang et al. (23)	China, Zhengzhou	Case-control	1,750/582	28.0/24.3	64.8/64.9	2010	5
Zhou and Li (36)	China, Kaifeng	Case-control, matched	162/245	20.4/20.3	63.0/67.0	2010	7
Zhai et al. (37)	China, Danzhou	Case-control, matched	582/582	30.3/38.4	64.9/61.0	2010	5
Xiong et al. (38)	China, Shenzhen	Case-control, matched	36/38	29.4/37.2	75.0/60.5	2010	7
Xiong et al. (39)	China, Shenzhen	Case-Control, matched	101/101	22.1/21.1	62.4/62.4	2010	7
Bai and Wu (40)	China, Nanyang	Case-control	94/337	37.2/45.6	56.4/51.0	2010	5
Du et al. (41)	China, Guangdong	Case-control	3,738/920,011	NA	64.4/66.6	2008 or 2010	6
Liu et al. (42)	China, Shantou	Case-control, matched	225/492	28.6/30.8	67.6/61.6	2010	6
Fu et al. (43)	China, Hainan	Case-control	663/624	29.8/38.0	66.5/62.7	2010	5
Zhang et al. (44)	China, Chongqing	Case-control	90/90	NA	67.8/62.2	2010	5
Xuan et al. (45)	China, Jilin	Case-control	50/50	24/24	NA	2010	7
Luo et al. (46)	China, Qiandongnan Miao and Dong Autonomous Prefecture	Case-control, matched	43/43	NA	62.8/NA	2009	8
Li and Gu (47)	China, Wuxi	Case-control	177/240	NA	63.8/NA	2009 or 2010	5
Ying et al. (48)	China, Lianyungang	Case-control	151/1,535	NA	/	2010	6
Lv et al. (49)	China, Shaoxing	Case-control	175/183	NA	70.9/66.1	2010	5
Du et al. (50)	China, Zhaoqing	Case-control	182/265	21/21.6	65.9/69.8	2009 or 2010	7
Wang et al. (12)	China, Liuzhou	Case-control	57/114	21.4/22.8	NA/68.4	2009	7
Mou et al. (51)	China, Shenzhen	Nested case-control	286/286	24.1/29.8	62.9/65.7	2009 or 2010	7
Duan et al. (52)	China, Zhengzhou	Nested case-control	65/37	23.5/22.6	60.0/62.2	2010	7
Xie et al. (53)	China, Huaihua	Case-control	80/262	26.1/24.5	50.0/49.6	2010	5
Zhibing (54)	China, Beijing	Case-control	56/56	56.7/51.6	57.1/62.5	2010	5
Li et al. (55)	China, Xi'an	Case-control	116/202	NA	71.6/63.4	2010	7
Yang et al. (56)	China, Wuhan	Case-control	50/100	NA	68.0/53.0	2009	6
Ruan et al. (57)	China, Xiamen	Case-control	31/69	28.8/37.2	NA	2010	6
Pan et al. (58)	China, Shanghai	Case-control	105/210	25.8/32.9	69.5/55.2	2010	6
Wu et al. (59)	China, Shanghai	Case-control, matched	73/146	23/25.4	63.0/64.4	2010	8
Cao et al. (60)	China, Anhui	Case-control	85/57	NA	NA	2008	6

### Association of EV 71 With Severe HFMD

**Figure 2:**
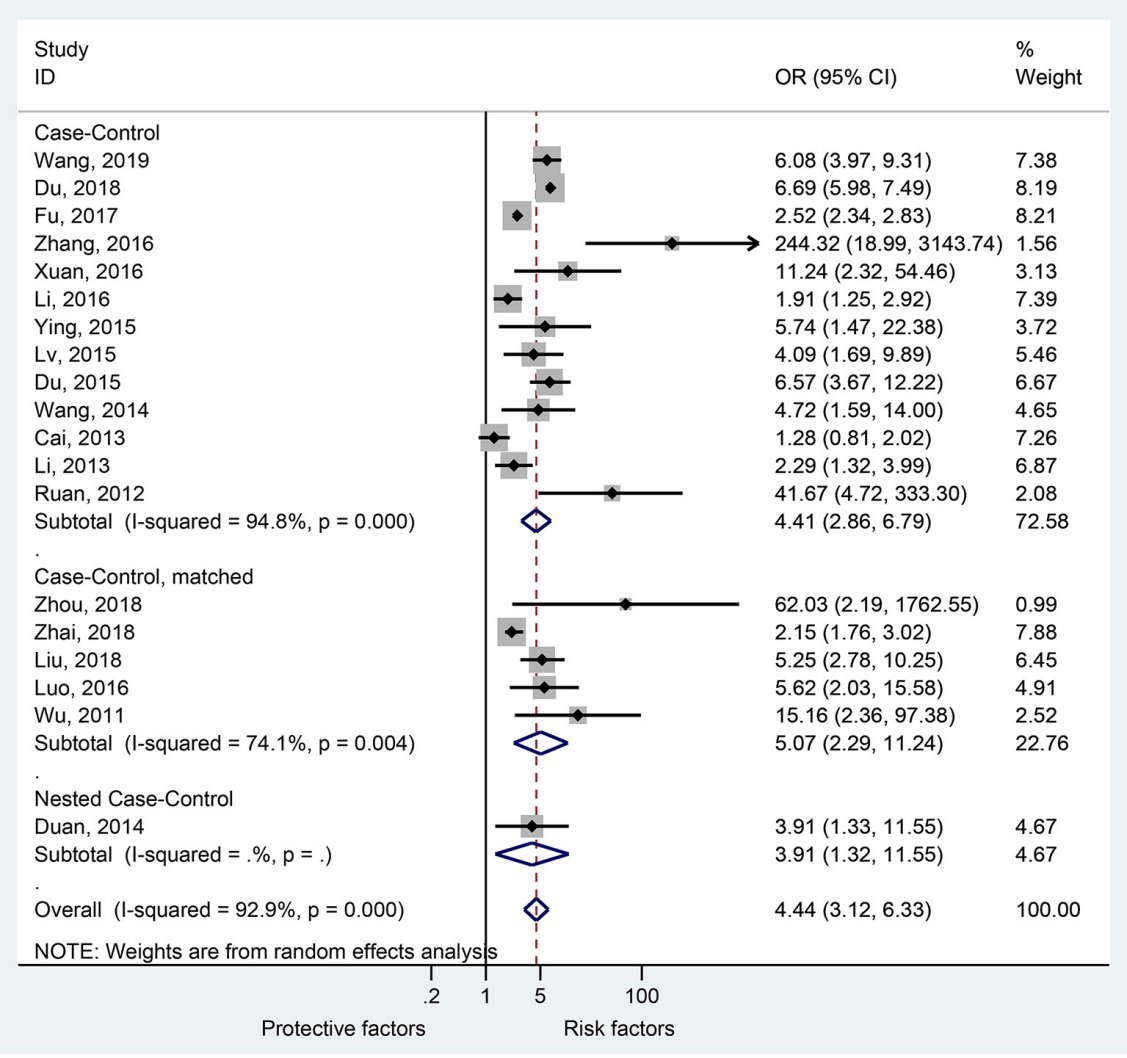
Forest plot for the association of enterovirus 71 with severe hand foot mouth disease.

Nineteen studies presented data regarding the association of EV 71 with severe HFMD. The overall meta-analysis showed that EV 71 is associated with higher odds of severe HFMD compared with other etiologic agents (OR = 4.44, 95%CI: 3.12–6.33, *p* < 0.001, *I*^2^ = 92.9%, *p*_heterogeneity_ < 0.001) (Figure 2). This association was observed irrespective of the type of study: case-control (OR = 4.41, 95%CI: 2.86–6.79, *p* < 0.001, *I*^2^ = 94.8%, *p*_heterogeneity_ < 0.001), matched case-control (OR = 5.07, 95%CI: 2.29–11.24, *p* < 0.001, *I*^2^ = 74.1%, *p*_heterogeneity_ = 0.004), and nested case-control (OR = 3.91, 95%CI: 1.32–11.55, *p* = 0.014).

The sensitivity analysis (Supplementary Figure 1A) showed that Du 2018 (41) influenced the results of the association of EV 71 with severe HFMD. It can be seen that the *I*^2^ decreased to 78.1% after Du 2018 (41) was removed (Supplementary Figure 1B).

### Demographic Factors

**Figure 3:**
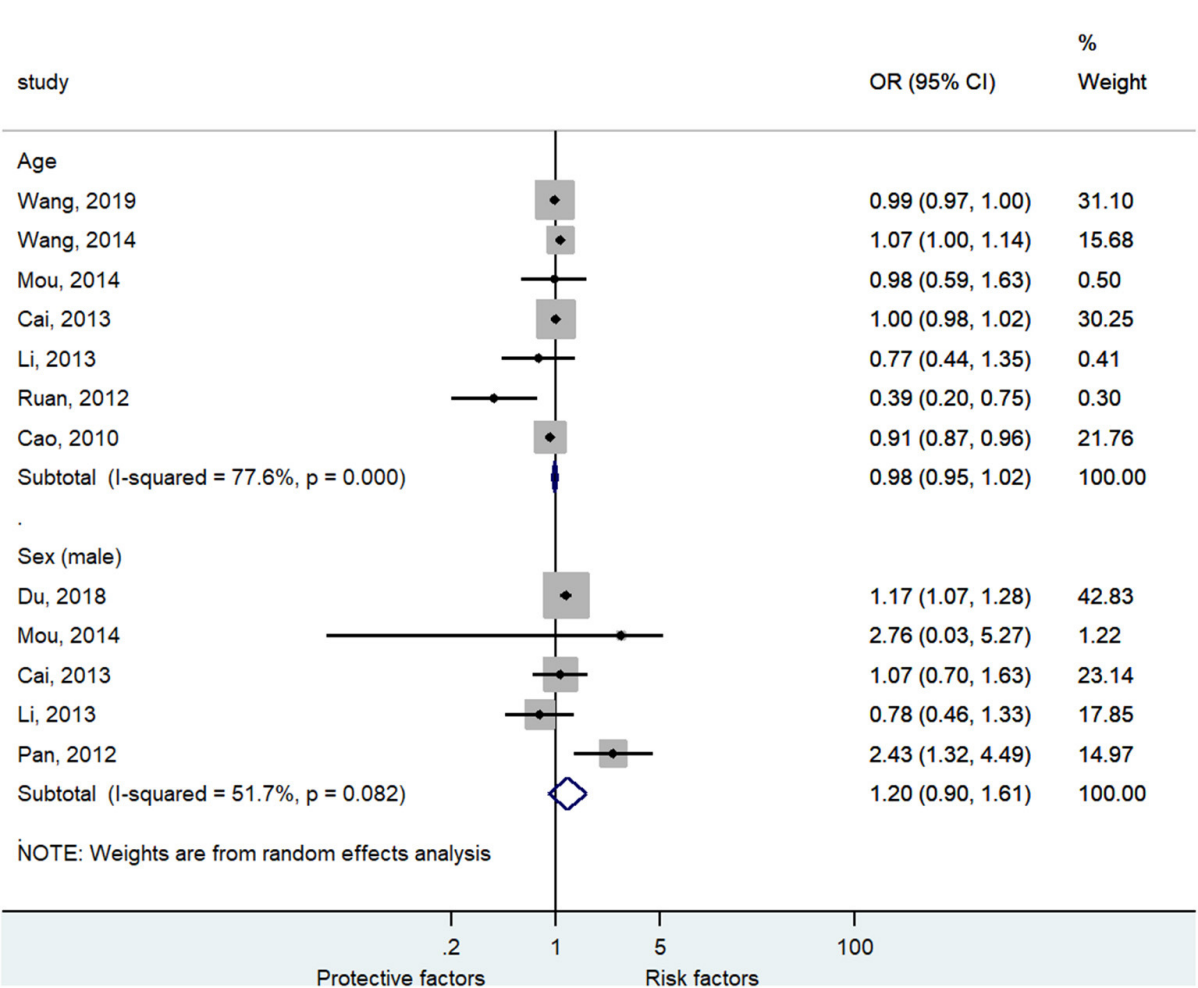
Forest plot for the association of age and sex with severe hand foot mouth disease.

Figure 3 and Supplementary Figure 2 shows that age (seven studies, OR = 0.98, 95%CI: 0.95–1.02, *p* = 0.35, *I*^2^ = 77.6%, *p*_heterogeneity_ < 0.001) and male sex (five studies, OR = 1.20, 95%CI: 0.90–1.61, *p* = 0.22, *I*^2^ = 51.7%, *p*_heterogeneity_ = 0.082) were not associated with severe HFMD.

### Socio-Economic Status and Lifestyle

**Figure 4:**
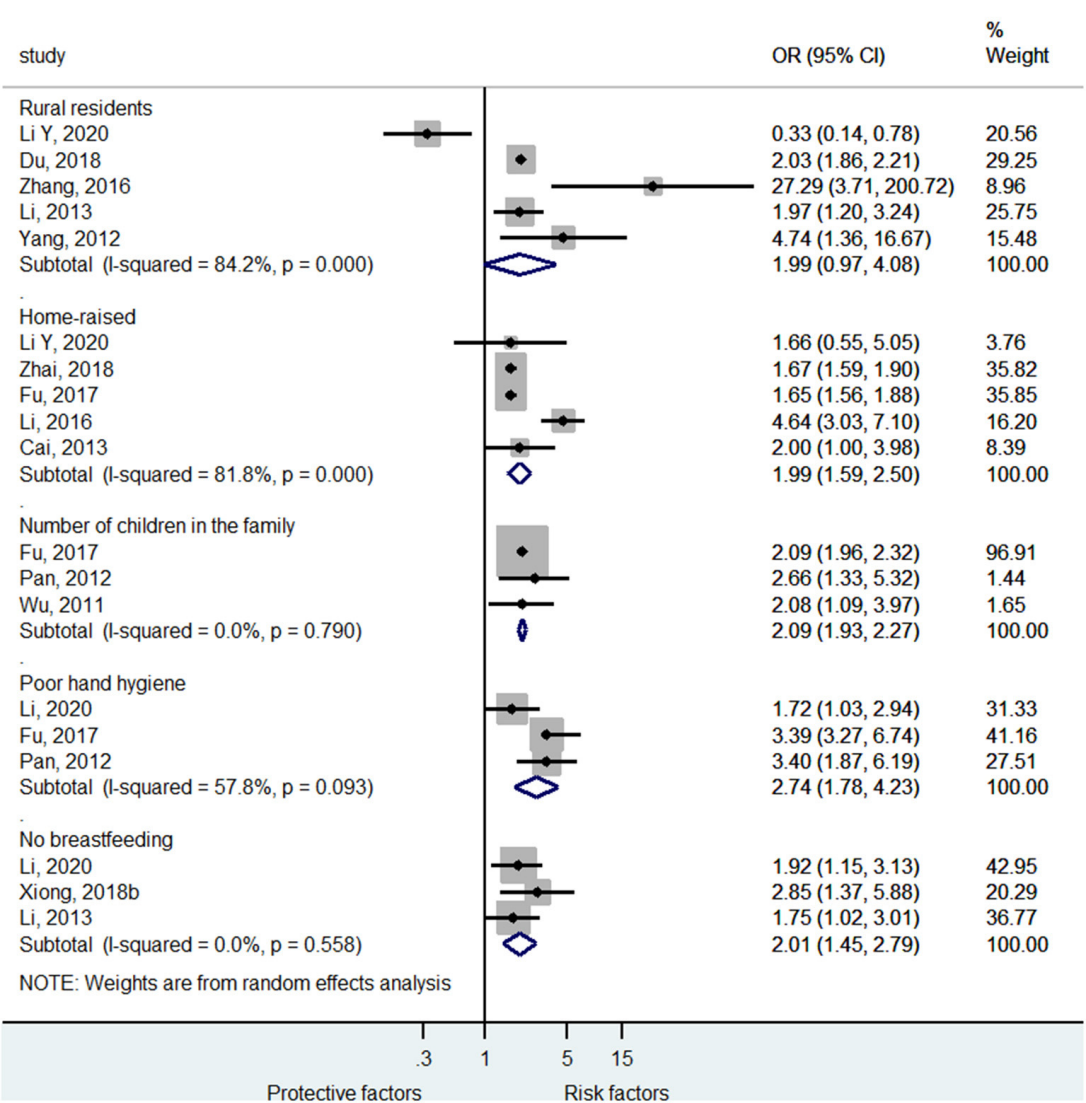
Forest plot for the association of lifestyle of life habits with severe hand foot mouth disease.

Figure 4 and Supplementary Figure 2 shows that rural residency (OR = 1.99, 95%CI: 0.97–4.08, *p* < 0.059, *I*^2^ = 84.2%, *p*_heterogeneity_ < 0.001) was not a risk factor for severe HFMD, while being home-raised (i.e., not frequenting kindergarten) (OR = 1.99, 95%CI: 1.59–2.50, *p* < 0.001, *I*^2^ = 81.8%, *p*_heterogeneity_ < 0.001), higher number of children in the family (OR = 2.09, 95%CI: 1.93–2.27, *p* < 0.001, *I*^2^ = 0.0%, *p*_heterogeneity_ = 0.790), poor hand hygiene (OR = 2.74, 95%CI: 1.78–4.23, *p* < 0.001, *I*^2^ = 57.8%, *p*_heterogeneity_ = 0.093), and no breastfeeding (OR = 2.01, 95%CI: 1.45–2.79, *p* < 0.001, *I*^2^ = 0.0%, *p*_heterogeneity_ = 0.558) were risk factors for severe HFMD.

### Clinical Pathway

**Figure 5:**
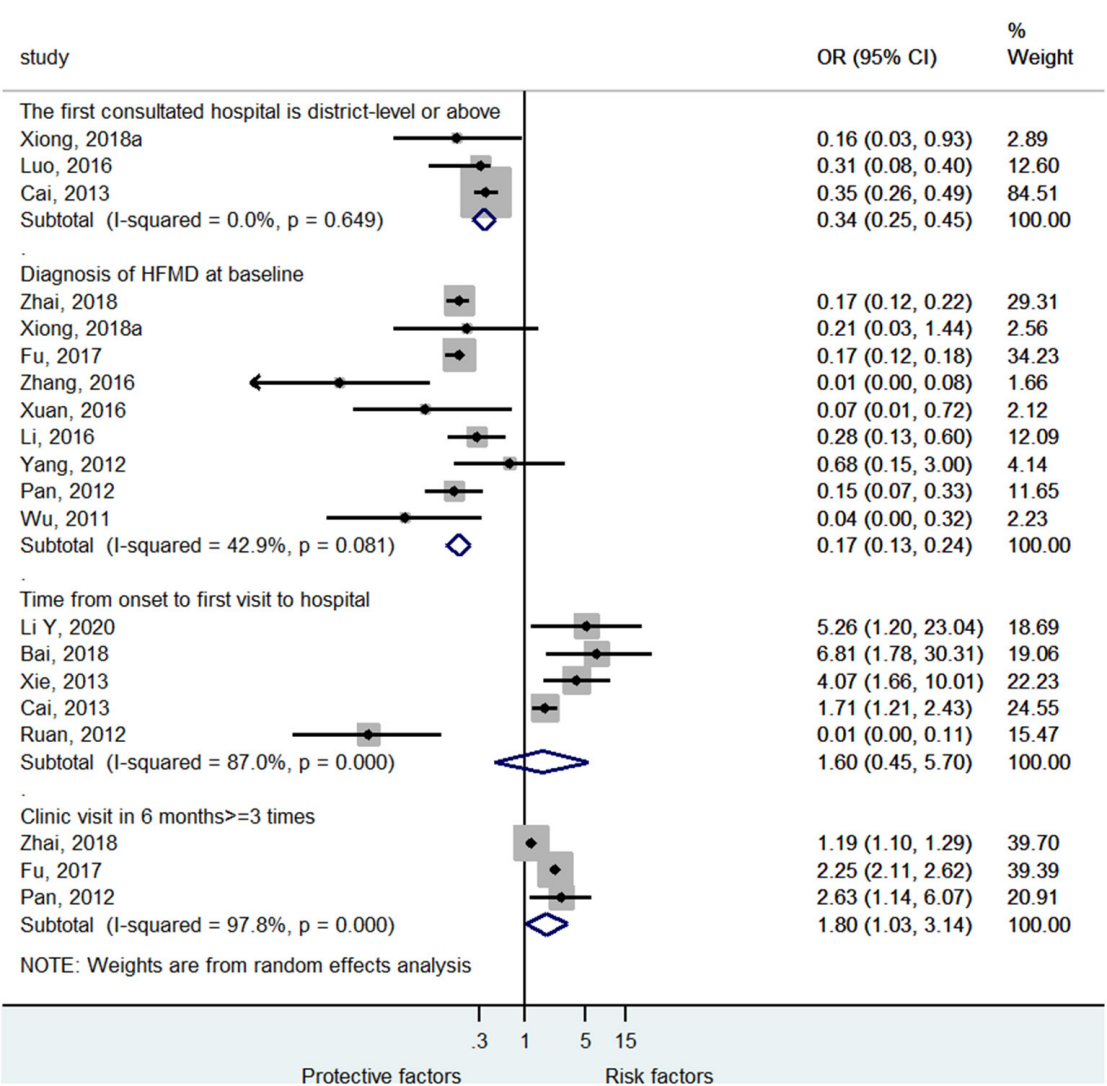
Forest plot for the association of the clinical pathway characteristics with severe hand foot mouth disease.

Among the clinical pathway factors that could influence the outcomes of HFMD, first consulting to a district-level or above hospital (OR = 0.34, 95%CI: 0.25–0.45, *p* < 0.001, *I*^2^ = 0.0%, *p*_heterogeneity_ = 0.649) and diagnosis of HFMD at baseline (OR = 0.17, 95%CI: 0.13–0.24, *p* < 0.001, *I*^2^ = 42.9%, *p*_heterogeneity_ = 0.081) were protective factor against severe HFMD (Figure 5; Supplementary Figure 2), while >3 visits at the clinic in the past 6 months was a risk factor for severe HFMD (OR = 1.80, 95%CI: 1.03–3.14, *p* = 0.037, *I*^2^ = 97.8%, *p*_heterogeneity_ < 0.001). The time from onset to the first visit at the hospital was not associated with severe HFMD (OR = 1.60, 95%CI: 0.45–5.70, *p* = 0.467, *I*^2^ = 87.0%, *p*_heterogeneity_ < 0.001).

### Symptoms

Supplementary Table 1 and Supplementary Figure 2 show that fever (OR = 3.23, 95%CI: 2.31–4.70, *p* < 0.001, *I*^2^ = 66.2%, *p*_heterogeneity_ < 0.001), long fever duration (OR = 5.36, 95%CI: 2.33–12.35, *p* = 0.001, *I*^2^ = 91.3%, *p*_heterogeneity_ < 0.001), vomiting (OR = 64.30, 95%CI: 5.53–747.80, *p* < 0.001, *I*^2^ = 48.1%, *p*_heterogeneity_ = 0.103), lethargy (OR = 8.89, 95%CI: 2.55–30.93, *p* = 0.001, *I*^2^ = 84.1%, *p*_heterogeneity_ < 0.001), leukocytosis (OR = 2.26, 95%CI: 1.42–3.61, *p* < 0.001, *I*^2^ = 87.5%, *p*_heterogeneity_ = 0.720), tremors/myoclonic jerks (OR = 5.20, 95%CI: 3.14–8.64, *p* < 0.001, *I*^2^ = 5.9%, *p*_heterogeneity_ < 0.001), and convulsions (OR = 12.64, 95%CI: 1.15–139.00, *p* = 0.038, *I*^2^ = 53.0%, *p*_heterogeneity_ = 0.145) were each associated with severe HFMD, while rash was not (OR = 1.40, 95%CI: 0.25–7.87, *p* = 0.704, *I*^2^ = 87.3%, *p*_heterogeneity_ < 0.001).

### Publication Bias

**Figure 6:**
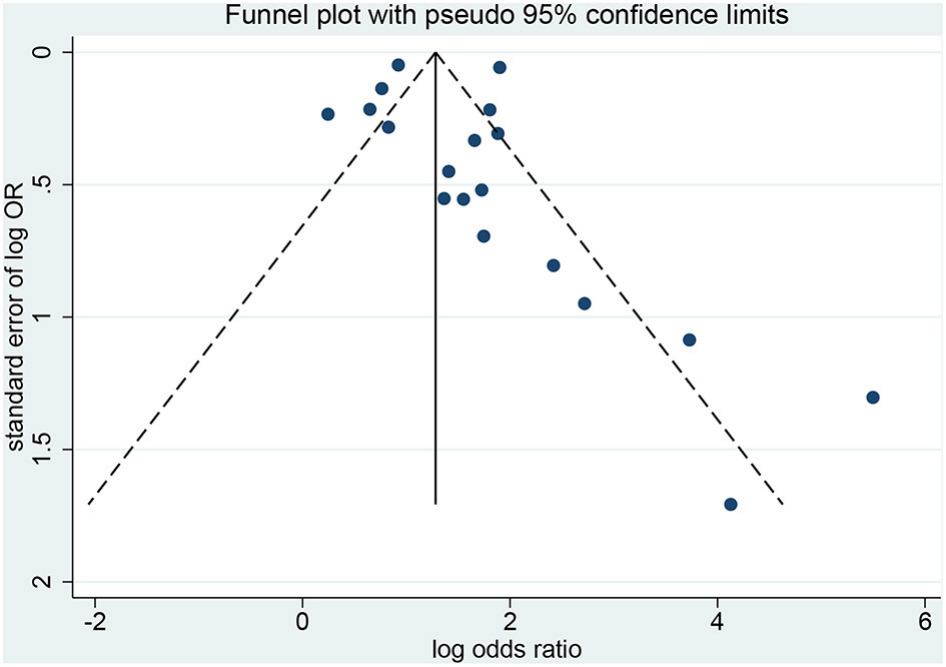
Funnel plot for publication bias.

**Figure 7:**
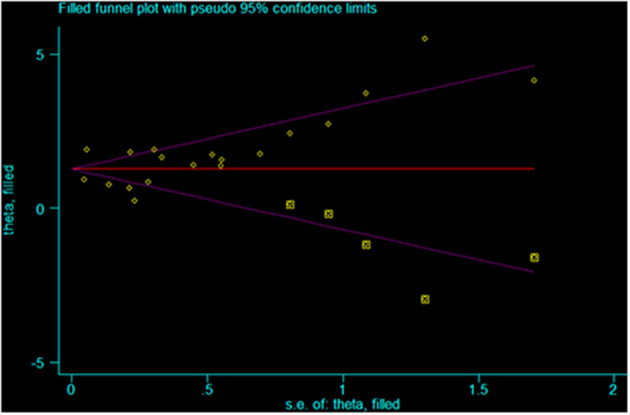
Filled funnel plot for publication bias.

The Egger test (*p* < 0.001) and the Begg test (*p* = 0.021) showed that there is publication bias (Figures 6, 7). Five studies would be needed to balance de publication bias (Figure 7).

## Discussion

Various risk factors for severe HFMD were reported in the literature (18–20, 61), but there is a lack of consensus. This study aimed to identify potential risk factors for severe HFMD. The results indicate that EV 71, lifestyle habits (rural, being home-raised, a higher number of children in the family, poor hand hygiene, and no breastfeeding), clinical pathway (frequent hospital visits), and symptoms are risk factors for severe HFMD in children, while early diagnosis and admission to higher-level hospitals are protective factors.

Previous meta-analyses examined HFMD (18, 19, 61). Indeed, Sun et al. (18) examined the risk factors for severe HFMD. Peng et al. (19) examined the risk factors for neurogenic pulmonary edema in children with severe HFMD. Fang et al. (62) examined the risk factors for severe HFMD. Ni et al. (61) investigated the association between risk factors and death from HFMD. Therefore, in an innovative view, the present study focused on the risk factors and the changes in disease severity (from mild to severe).

EV 71 is a well-known risk factor for severe HFMD (4, 63–65), and it was confirmed once again. This EV serotype is the cause of HFMD outbreaks in the Asia-Pacific region, although it has been reported to be the cause of outbreaks in Europe and North America in the 1970s (66). This meta-analysis also showed that rural inhabitants are at higher risk of severe HFMD, probably because of poorer hygiene, lower education, lower economic status, and closer proximity to farm animals (e.g., living on a farm) which are supported by previous studies (67–69). In addition, being home-raised, a higher number of children in the family, and poor hand hygiene are already known to be associated with higher risks of transmission of various pediatric diseases, as previously observed (44, 70, 71). On the other hand, a study showed that children attending daycare centers were at higher risk of severe HFMD (72). No breastfeeding is associated with a lower immune status in some children and could lead to more severe HFMD. Some previous studies showed that exclusive breastfeeding protected against HFMD (18, 73), supporting this meta-analysis.

As for any disease, early diagnosis and management are associated with better outcomes, as observed for HFMD (67, 74, 75). Higher-level hospitals are more likely to have experienced physicians with more awareness for the fast and accurate diagnosis of HFMD, as well as for proper management (35). In this study, the diagnosis of HFMD as the first diagnosis and higher-level hospitals were protective factors against severe HFMD. Frequent visits to the hospital might lead to higher nosocomial exposure to hospitals' pathogens, frailer constitution, or decreased awareness from the healthcare professionals toward children and their parents consulting too often. This will have to be examined more closely in future studies.

Symptom severity has already been associated with severe HFMD in previous meta-analyses and reviews. Our previous study illustrated a causal relationship between EV 71 infection and severe HFMD risk, the symptoms of critical illness including autokinetic eyeball, eyeball ataxia, severe coma, respiratory rhythm abnormality, ataxic respiration, absence of pharyngeal reflex, ultrahyperpyrexia (>40°C), excessive tachycardia (>220 bpm), pulmonary edema, and/or hemorrhage and refractory shock; and four indicators with time-correlation with half of the symptoms above, including CRT (capillary refill time) extension, tachycardia, hyperventilation, and nystagmus, which occurred before the above-mentioned critical illness symptoms and also indicated the risk of critical illness (76). Sun et al. (18) showed that fever >3 days and vomiting were associated with severe HFMD. Peng et al. (19) showed that limb tremors, vomiting, abnormal respiration, atypical rash, and drowsiness were associated with HFMD-associated pulmonary edema. The present study also showed that fever, long fever duration, vomiting, lethargy, leukocytosis, tremors/myoclonic jerks, and convulsions were risk factors for severe HFMD. Nevertheless, this study did not examine the risk factors of various complications of HFMD.

This study has limitations that must be considered when analyzing the results. First, although the eligibility criteria did not restrict the country and western databases (PubMed, Embase, and the Cochrane Library) were queried, all included studies were from China. It is probably because HFMD reaches epidemic proportions in some regions of China nearly every year and that EV 71 is particularly present and associated with severe forms of HFMD (4, 63–65). Western countries like the United States of America only have episodic HFMD epidemic events once every few years (3). Furthermore, heterogeneity was observed among the studies because of different study designs, different diagnostic criteria for severe HFMD, and different confounding factors included in the multivariable models. Of note, for the analysis of the association between EV 71 and severe HFMD, excluding Du 2018 (41) decreased heterogeneity. The possible reason is that the sample size of that study is too large compared with the other studies. Finally, publication bias was observed and indicated that positive-findings studies were overrepresented compared with the negative-findings ones. According to Figure 7, five studies would be needed to balance de publication bias.

In conclusion, this meta-analysis indicates that EV 71 infection, lifestyle habits (rural, being home-raised, a higher number of children in the family, poor hand hygiene, and no breastfeeding), clinical pathway (frequent hospital visits), and symptoms are risk factors for severe HFMD in children, while early diagnosis and admission to higher-level hospitals are protective factors. Age, sex, and time from onset to admission are not associated with HFMD severity.

## Data Availability Statement

The original contributions presented in the study are included in the article/Supplementary Material, further inquiries can be directed to the corresponding author/s.

## Author Contributions

PL and YH conceived and coordinated the study, designed, performed and analyzed the experiments, and wrote the paper. DZ, DH, and SY carried out the data collection, data analysis, and revised the paper. All authors reviewed the results and approved the final version of the manuscript.

## Funding

This work was supported by the National Natural Science Foundation of China (No. 81801206), the Medical Science and Technology Research Foundation of Guangdong, China (No. A2019373), Guangdong Natural Science Foundation (No. 2020A1515010014), Science and Technology Key Project for People's Livelihood of Guangzhou, China (No. 201803010026), and the Innovative Project of Children's Research Institute, Guangzhou Women and Children's Medical Center, China (Nos. Pre-NSFC-2018-004, Pre-NSFC-2018-008, Pre-NSFC-2019-002, and Pre-NSFC-2019-007). The funders had no role in study design, data collection, and analysis, decision to publish, or preparation of the manuscript.

## Conflict of Interest

The authors declare that the research was conducted in the absence of any commercial or financial relationships that could be construed as a potential conflict of interest.

## Publisher's Note

All claims expressed in this article are solely those of the authors and do not necessarily represent those of their affiliated organizations, or those of the publisher, the editors and the reviewers. Any product that may be evaluated in this article, or claim that may be made by its manufacturer, is not guaranteed or endorsed by the publisher.

## References

[1] Sarma N. Hand, foot, and mouth disease: current scenario and Indian perspective. Indian J Dermatol Venereol Leprol. (2013) 79:165–75. 10.4103/0378-6323.107631 23442455

[2] Aswathyraj S Arunkumar G Alidjinou EK Hober D. Hand, foot and mouth disease (HFMD): emerging epidemiology and the need for a vaccine strategy. Med Microbiol Immunol. (2016) 205:397–407. 10.1007/s00430-016-0465-y 27406374

[3] Repass GL Palmer WC Stancampiano FF. Hand, foot, and mouth disease: identifying and managing an acute viral syndrome. Cleve Clin J Med. (2014) 81:537–43. 10.3949/ccjm.81a.13132 25183845

[4] World Health Organization. A Guide to Clinical Management and Public Health Response for Hand, Foot and Mouth Disease (HFMD). Geneva: World Health Organization (2018). Available online at: https://iris.wpro.who.int/bitstream/handle/10665.1/5521/9789290615255_eng.pdf

[5] Xiao Y Zhou J Zhang H Ding C Shi P. Epidemiological and aetiological characteristics of hand, foot and mouth disease cases 2011–2017 in Yixing, China. Infect Dis (Lond). (2018) 50:859–61. 10.1080/23744235.2018.1493217 30045640

[6] Qi L Tang W Zhao H Ling H Su K Zhao H . Epidemiological characteristics and spatial-temporal distribution of hand, foot, and mouth disease in Chongqing, China, 2009-2016. Int J Environ Res Public Health. (2018) 15:270. 10.3390/ijerph15020270 29401726 PMC5858339

[7] Chan LG Parashar UD Lye MS Ong FG Zaki SR Alexander JP . Deaths of children during an outbreak of hand, foot, and mouth disease in Sarawak, Malaysia: clinical and pathological characteristics of the disease. For the Outbreak Study Group. Clin Infect Dis. (2000) 31:678–83. 10.1086/314032 11017815

[8] Ho M Chen ER Hsu KH Twu SJ Chen KT Tsai SF . An epidemic of enterovirus 71 infection in Taiwan. Taiwan Enterovirus Epidemic Working Group. N Engl J Med. (1999) 341:929–35. 10.1056/NEJM199909233411301 10498487

[9] Xu W Jiang L Thammawijaya P Thamthitiwat S. Hand, foot and mouth disease in Yunnan Province, China, 2008–2010. Asia Pac J Public Health. (2015) 27:NP769–77. 10.1177/1010539511430523 22199158

[10] Kit LS. Emerging and re-emerging diseases in Malaysia. Asia Pac J Public Health. (2002) 14:6–8. 10.1177/101053950201400103 12597511

[11] Messacar K Spence-Davizon E Osborne C Press C Schreiner TL Martin J. Clinical characteristics of enterovirus A71 neurological disease during an outbreak in children in Colorado, USA, in 2018: an observational cohort study. Lancet Infect Dis. (2020) 20:230–9. 10.1016/S1473-3099(19)30632-2 31859216 PMC11284833

[12] Wang Y He X Zhao J. Mortality and death cases of hand foot and mouth disease reported in China, 2008–2010. Dis Surveil. (2011) 26:424–6.

[13] Esposito S Principi N. Hand, foot and mouth disease: current knowledge on clinical manifestations, epidemiology, aetiology and prevention. Eur J Clin Microbiol Infect Dis. (2018) 37:391–8. 10.1007/s10096-018-3206-x 29411190

[14] Hu P Hou S Du PF Li JB Ye Y. Paroxysmal supraventricular tachycardia in an infant with hand, foot, and mouth disease. Ann Dermatol. (2012) 24:200–2. 10.5021/ad.2012.24.2.200 22577272 PMC3346912

[15] Yang SD Li PQ Huang YG Li W Ma LZ Wu L . Factors associated with fatal outcome of children with enterovirus A71 infection: a case series. Epidemiol Infect. (2018) 146:788–98. 10.1017/S0950268818000468 29526169 PMC9134374

[16] Li P Wang N Ma W Gao Y Yang S. Hand, foot, and mouth disease in mainland China. Lancet Infect Dis. (2014) 14:1042. 10.1016/S1473-3099(14)70974-0 25444403

[17] Ma H He F Wan J Jin D Zhu L Liu X . Glucocorticoid and pyrazolone treatment of acute fever is a risk factor for critical and life-threatening human enterovirus 71 infection during an outbreak in China, 2008. Pediatr Infect Dis J. (2010) 29:524–9. 10.1097/INF.0b013e3181cdd178 20104199

[18] Sun BJ Chen HJ Chen Y An XD Zhou BS. The risk factors of acquiring severe hand, foot, and mouth disease: a meta-analysis. Can J Infect Dis Med Microbiol. (2018) 2018:2751457. 10.1155/2018/2751457 30046361 PMC6038695

[19] Peng L Luo R Jiang Z. Risk factors for neurogenic pulmonary edema in patients with severe hand, foot, and mouth disease: a meta-analysis. Int J Infect Dis. (2017) 65:37–43. 10.1016/j.ijid.2017.09.020 28970089

[20] Shi C Liu J Shi P Ji H Shen Y Qian YH. Epidemiological characteristics and influential factors of hand, foot, and mouth disease reinfection in Wuxi, China, 2008–2016. BMC Infect Dis. (2018) 18:472. 10.1186/s12879-018-3385-1 30231857 PMC6146628

[21] Moher D Liberati A Tetzlaff J Altman DG Group P. Preferred reporting items for systematic reviews and meta-analyses: the PRISMA statement. PLoS Med. (2009) 6:e1000097. 10.1371/journal.pmed.1000097 19621072 PMC2707599

[22] Selcuk AA. A guide for systematic reviews: PRISMA. Turk Arch Otorhinolaryngol. (2019) 57:57–8. 10.5152/tao.2019.4058 31049257 PMC6461330

[23] Wang B Feng H Huang P Dang D Zhao J Yi J . Developing a nomogram for risk prediction of severe hand-foot-and-mouth disease in children. Indian J Pediatr. (2019) 86:365–70. 10.1007/s12098-019-02898-4 30798415

[24] National Health Commission of the People's Republic of China. Diagnosis and treatment guideline on hand, foot and mouth disease 2018. Chin J Clin Infect Dis. (2018) 11:161–6.

[25] Lo CK Mertz D Loeb M. Newcastle-Ottawa Scale: comparing reviewers' to authors' assessments. BMC Med Res Methodol. (2014) 14:45. 10.1186/1471-2288-14-45 24690082 PMC4021422

[26] DerSimonian R Laird N. Meta-analysis in clinical trials. Control Clin Trials. (1986) 7:177–88. 10.1016/0197-2456(86)90046-2 3802833

[27] Ades AE Lu G Higgins JP. The interpretation of random-effects meta-analysis in decision models. Med Decis Making. (2005) 25:646–54. 10.1177/0272989X05282643 16282215

[28] Higgins J Thomas J Chandler J Cumpston M Li T Page M. Cochrane Handbook for Systematic Reviews of Interventions version 6.0 (updated July 2019). London: Cochrane Collaboration (2019). 10.1002/9781119536604

[29] Higgins JP Thompson SG Deeks JJ Altman DG. Measuring inconsistency in meta-analyses. BMJ. (2003) 327:557–60. 10.1136/bmj.327.7414.557 12958120 PMC192859

[30] Tobias A. Assessing the influence of a single study in meta-analysis. Stata Tech Bull. (1999) 47:15–7.

[31] Egger M Davey Smith G Schneider M Minder C. Bias in meta-analysis detected by a simple, graphical test. BMJ. (1997) 315:629–34. 10.1136/bmj.315.7109.629 9310563 PMC2127453

[32] Begg CB Mazumdar M. Operating characteristics of a rank correlation test for publication bias. Biometrics. (1994) 50:1088–101. 10.2307/2533446 7786990

[33] Duval S Tweedie R. Trim and fill: a simple funnel-plot-based method of testing and adjusting for publication bias in meta-analysis. Biometrics. (2000) 56:455–63. 10.1111/j.0006-341X.2000.00455.x 10877304

[34] Li Y Xu X. Analysis of risk factors in critical cases of hand-foot-mouth disease. Med Recapitul. (2020). 26:4143–6; 4152.

[35] Liu J Qi J. Prevalence and management of severe hand, foot, and mouth disease in Xiangyang, China, from 2008 to 2013. Front Pediatr. (2020) 8:323. 10.3389/fped.2020.00323 32754560 PMC7366859

[36] Zhou L Li X. Analysis of risk factors in severe cases of hand foot mouth disease. China Med Eng. (2018) 26.

[37] Zhai J Lin L Mai L Fu X. Risk factors of severe hand, foot and mouth disease in Danzhou of China. Chin J Viral Dis. (2018) 8:44–9.

[38] Xiong T Meng Y Liu J Huang J Zhao R Wu T. Analysis on risk factors of severe cases with hand foot mouth disease infected by Coxsackievirus A6. Jiangsu J Prev Med. (2018). [Epub ahead of print].

[39] Xiong T Meng Y Yu G Huang J Zhao R Wei S. Risk factors of severe cases with hand-foot-mouth disease infected by EV-A71. China Trop Med. (2018). 18:242–4.

[40] Bai T Wu Z. Analysis on risk factors for severe hand, foot, and mouth disease in a tertiary hospital, Nanyang city 2016 and 2017. Prev Med Trib. (2018) 24:354–6.

[41] Du Z Lawrence WR Zhang W Zhang D Yu S Hao Y. Bayesian spatiotemporal analysis for association of environmental factors with hand, foot, and mouth disease in Guangdong, China. Sci Rep. (2018) 8:15147. 10.1038/s41598-018-33109-3 30310172 PMC6181968

[42] Liu C Wang K Lin N Cai J Cui B Wu B. Risk factors of severe hand, foot and mouth disease in Shantou, China: a case-control study. J Infect Dev Ctries. (2018) 12:359–64. 10.3855/jidc.9845 31865299

[43] Fu T Yang J Lin W Lie X. Risk factors of severe hand-foot-mouth disease in the southern area of Hainan Province. Acad J Chin PLA Med Sch. (2017) 38:132–5. 10.1177/1010539515579123 25850695

[44] Zhang D Li Z Zhang W Guo P Ma Z Chen Q . Hand-washing: the main strategy for avoiding hand, foot and mouth disease. Int J Environ Res Public Health. (2016) 13:610. 10.3390/ijerph13060610 27322307 PMC4924067

[45] Xuan S Deng L Zhao Q Lu X Chen L. Analysis for the risk factors of severe hand foot mouth disease cases in Jilin province. Chin J Public Health Eng. (2016) 15:250–2.

[46] Luo T Shi D Deng M Shan Z Liu J. Epidemiological characteristics of severe cases of hand foot mouth disease in Qiandongnan Prefecture in 2014. J Appl Prev Med. (2016) 22.

[47] Li J Gu Y. The epidemiological characteristics and risk factors of severe hand foot- and mouth-disease in Xishan District of Wuxi City between 2011 and 2014. Mod Prevent Med. (2016) 43:972–5.

[48] Ying L. HFMD severe cases'risk factors analyses of Lianyungang city. J Med Pest Control. (2015) 31:803–5.

[49] Lv B Mo W Huang C Zhang M Ma A He J. Analysis of risk factors of severe hand, foot and mouth disease. Chin J Prim Med Pharm. (2015) 22:344–8.30046361

[50] Du W Wang L Xia Q. Analysis of risk factors in severe cases of hand foot mouth disease. Pract Prev Med. (2015) 22.

[51] Mou J Dawes M Li Y He Y Ma H Xie X . Severe hand, foot and mouth disease in Shenzhen, South China: what matters most? Epidemiol Infect. (2014) 142:776–88. 10.1017/S0950268813001453 23809877 PMC9151125

[52] Duan G Yang H Shi L Sun W Sui M Zhang R . Serum inflammatory cytokine levels correlate with hand-foot-mouth disease severity: a nested serial case-control study. PLoS ONE. (2014) 9:e112676. 10.1371/journal.pone.0112676 25391156 PMC4229228

[53] Xie Z. Risk factors of severe hand foot mouth disease in Huaihua City. J Clin Res. (2013) 30.

[54] Zhibing X. Analysis of related risk factors in severe cases of hand, foot and mouth disease in Huaihua City. (2013). 10.3969/j.issn.1671-7171.2013.09.055

[55] Li Y Dang S Deng H Wang W Jia X Gao N . Breastfeeding, previous Epstein-Barr virus infection, Enterovirus 71 infection, and rural residence are associated with the severity of hand, foot, and mouth disease. Eur J Pediatr. (2013) 172:661–6. 10.1007/s00431-013-1939-1 23344210

[56] Yang X Zhou X Kong D Yu B. Logistic regression analysis on the influencing factors of severe cases of HFMD in Wuhan City. J Pub Health Prev Med. (2012) 23:15–7.

[57] Ruan J Liang Y Huang J Su C. Case-control study of risk factors for severe hand-foot-mouth disease. Chin J Zoon. (2012) 28:857–60.

[58] Pan H Zheng YX Mao SH Hu JY Zheng Y Li J . [A case-control study on risk factors that associated with severe hand-foot-mouth disease in Shanghai]. Zhonghua Liu Xing Bing Xue Za Zhi. (2012) 33:763–7. 22967324

[59] Wu H Zheng Y Mao S Gu B Pan H. Study on risk factors associated with severe case of hand-foot-mouth disease in Shanghai. J Environ Occup Med. (2011) 28:257–61.22967324

[60] Cao M Liu H Wan J Zhu L. An case-control study of severe case of Hand-Foot-and-mouth disease(EV71) in Fuyang City, Anhui Province. Anhui J Prev Med. (2010) 16:19–20.

[61] Ni XF Li X Xu C Xiong Q Xie BY Wang LH . Risk factors for death from hand-foot-mouth disease: a meta-analysis. Epidemiol Infect. (2020) 148:e44. 10.1017/S0950268819002279 32102711 PMC7058831

[62] Xing W Liao Q Viboud C Zhang J Sun J Wu JT . Hand, foot, and mouth disease in China, 2008–12: an epidemiological study. Lancet Infect Dis. (2014) 14:308–18. 10.1016/S1473-3099(13)70342-6 24485991 PMC4035015

[63] Fan H Fu YS Shan J Shi C Zhang XF Huo X . [Surveillance on the epidemiological and etiological characteristics of hand-foot-mouth disease during the outbreaks in three cities of Jiangsu province, 2012–2015]. Zhonghua Liu Xing Bing Xue Za Zhi. (2016) 37:1608–14. 10.3760/cma.j.issn.0254-6450.2016.12.011 27998408

[64] Liu MY Liu J Lai W Luo J Liu Y Vu GP . Characterization of enterovirus 71 infection and associated outbreak of hand, foot, and mouth disease in Shawo of China in 2012. Sci Rep. (2016) 6:38451. 10.1038/srep38451 27941929 PMC5150535

[65] Yan XF Gao S Xia JF Ye R Yu H Long JE. Epidemic characteristics of hand, foot, and mouth disease in Shanghai from 2009 to 2010: enterovirus 71 subgenotype C4 as the primary causative agent and a high incidence of mixed infections with coxsackievirus A16. Scand J Infect Dis. (2012) 44:297–305. 10.3109/00365548.2011.634433 22176514

[66] Sabanathan S Tan le V Thwaites L Wills B Qui PT Rogier van Doorn H. Enterovirus 71 related severe hand, foot and mouth disease outbreaks in South-East Asia: current situation and ongoing challenges. J Epidemiol Community Health. (2014) 68:500–2. 10.1136/jech-2014-203836 24652348 PMC4033151

[67] Wang Y Zhao H Ou R Zhu H Gan L Zeng Z . Epidemiological and clinical characteristics of severe hand-foot-and-mouth disease (HFMD) among children: a 6-year population-based study. BMC Public Health. (2020) 20:801. 10.1186/s12889-020-08961-6 32460823 PMC7254654

[68] Qin L Dang D Wang X Zhang R Feng H Ren J . Identification of immune and metabolic predictors of severe hand-foot-mouth disease. PLoS ONE. (2019) 14:e0216993. 10.1371/journal.pone.0216993 31120941 PMC6532886

[69] Zhang D Li R Zhang W Li G Ma Z Chen X . A case-control study on risk factors for severe hand, foot and mouth disease. Sci Rep. (2017) 7:40282. 10.1038/srep40282 28084311 PMC5233949

[70] Guo N Ma H Deng J Ma Y Huang L Guo R . Effect of hand washing and personal hygiene on hand food mouth disease: a community intervention study. Medicine (Baltimore). (2018) 97:e13144. 10.1097/MD.0000000000013144 30572426 PMC6320109

[71] Huang WC Shih WL Yang SC Yen TY Lee JT Huang YC . Predicting severe enterovirus 71 infection: age, comorbidity, and parental behavior matter. J Microbiol Immunol Infect. (2017) 50:10–6. 10.1016/j.jmii.2014.11.013 25678038

[72] Suzuki Y Taya K Nakashima K Ohyama T Kobayashi JM Ohkusa Y . Risk factors for severe hand foot and mouth disease. Pediatr Int. (2010) 52:203–7. 10.1111/j.1442-200X.2009.02937.x 19663940

[73] Lin H Sun L Lin J He J Deng A Kang M . Protective effect of exclusive breastfeeding against hand, foot and mouth disease. BMC Infect Dis. (2014) 14:645. 10.1186/s12879-014-0645-6 25471294 PMC4273484

[74] Chen S Yi K Chen X Li L Tan X. A simple scoring system for quick, accurate, and reliable early diagnosis of hand, foot, and mouth disease. Med Sci Monit. (2018) 24:8627–38. 10.12659/MSM.911736 30487478 PMC6282650

[75] Ooi MH Wong SC Mohan A Podin Y Perera D Clear D . Identification and validation of clinical predictors for the risk of neurological involvement in children with hand, foot, and mouth disease in Sarawak. BMC Infect Dis. (2009) 9:3. 10.1186/1471-2334-9-3 19152683 PMC2637878

[76] Yang SD Li PQ Li YM Li W Lai WY Zhu CP . Clinical manifestations of severe enterovirus 71 infection and early assessment in a Southern China population. BMC Infect Dis. (2017) 17:153. 10.1186/s12879-017-2228-9 28212620 PMC5316173

