# Supplementary material for: Risk Factors for Severe Hand-Foot-Mouth Disease in China: A Systematic Review and Meta-Analysis
Source: Front Pediatr. 2021 Nov 10;9:716039. doi: 10.3389/fped.2021.716039 (PMC8631475; doi:10.3389/fped.2021.716039)
Supplement: Supplementary file 1 [file Data_Sheet_1.docx]

**Supplementary Table S1.** **Association of different symptoms with disease severity**

| Symptoms | No. of studies | OR | 95%CI | *p* | I^2^ | *p*_heterogenity_ |
| --- | --- | --- | --- | --- | --- | --- |
| Fever | 15 | 3.23 | 2.31-4.70 | <0.001 | 66.2 | <0.001 |
| Long fever duration | 7 | 5.36 | 2.33-12.35 | 0.001 | 91.3 | <0.001 |
| Vomit | 5 | 64.3 | 5.53-747.8 | <0.001 | 48.1 | 0.103 |
| Lethargy | 5 | 8.89 | 2.55-30.93 | 0.001 | 84.1 | 0<0.001 |
| Leukocytosis | 6 | 2.26 | 1.42-3.61 | <0.001 | 87.5 | 0<0.001 |
| Tremors/myoclonic jerks | 4 | 5.2 | 3.14-8.64 | <0.001 | 5.9 | 0.364 |
| Convulsion | 2 | 12.64 | 1.15-139 | 0.038 | 53 | 0.145 |
| Rash | 4 | 1.4 | 0.25-7.87 | 0.704 | 87.3 | <0.001 |

**A**

**
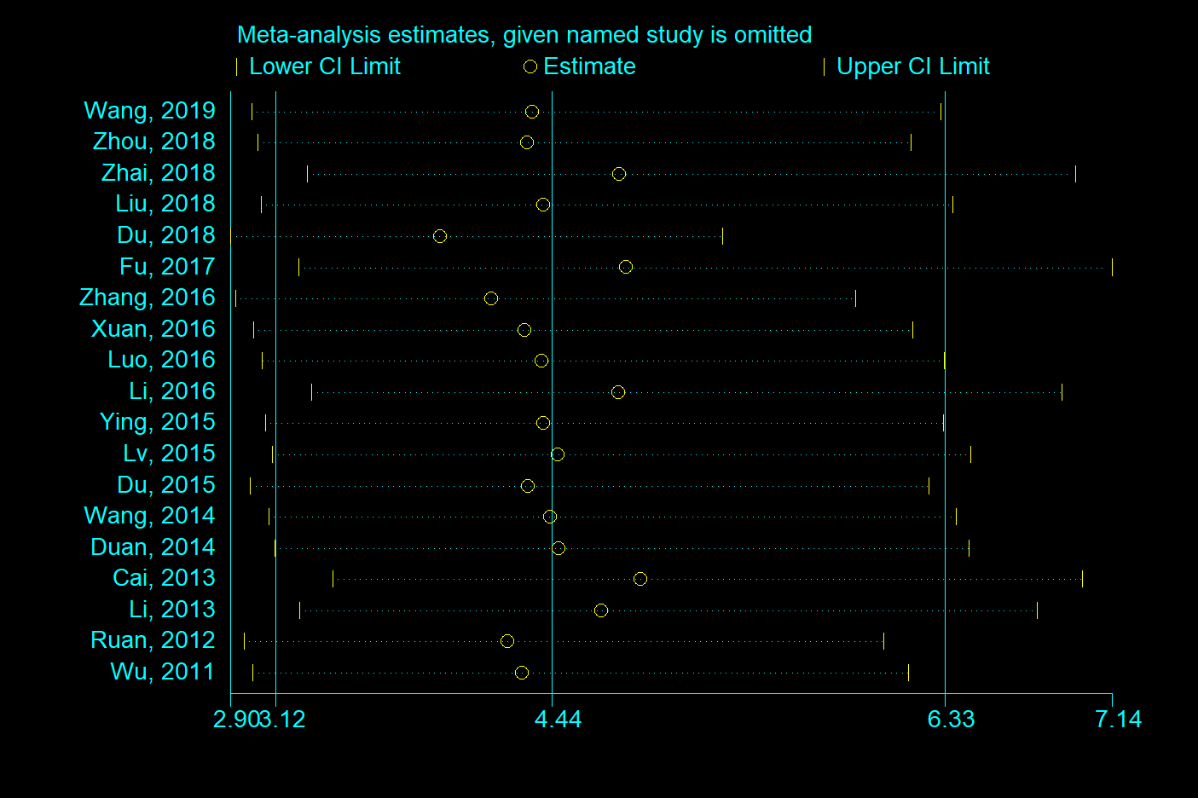
**

**B**

**
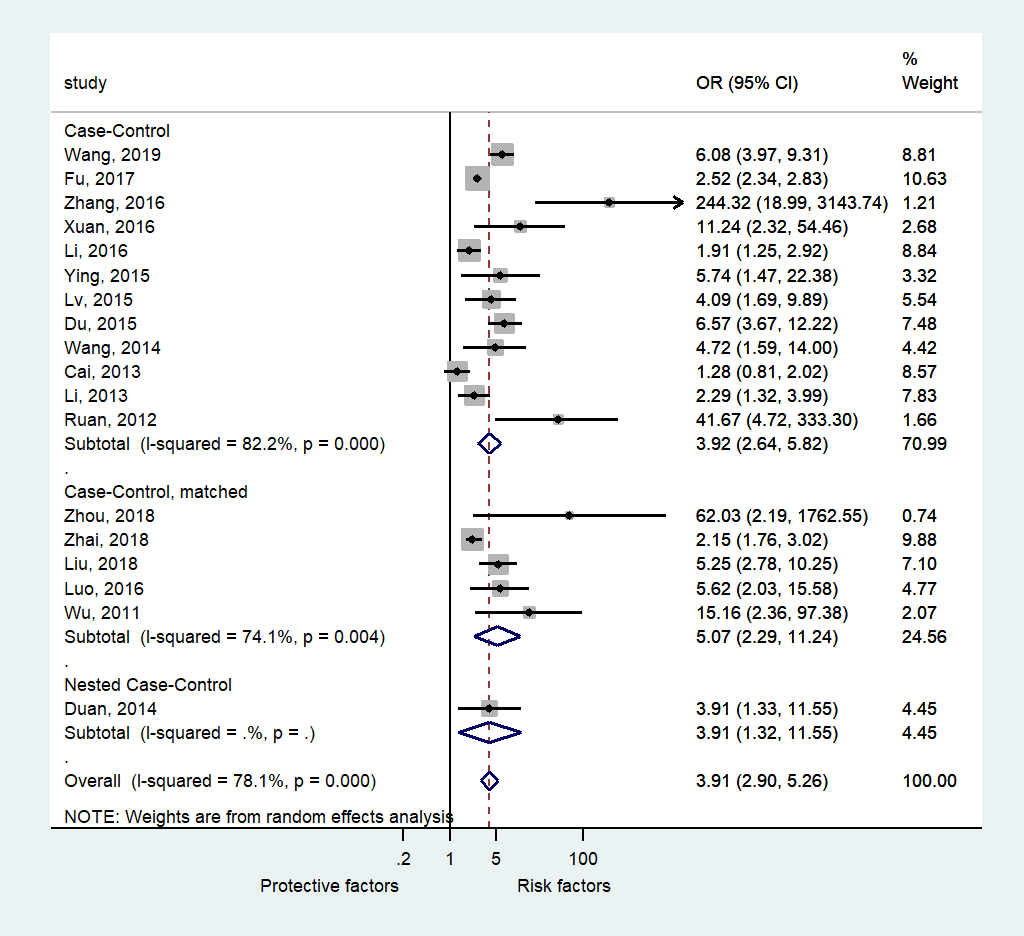
**

**Supplementary Figure S1.** (A) Sensitivity analysis for association of EV 71 with severe HFMD. (B) Forest plot for the association of enterovirus 71 with severe hand foot mouth disease after exclusion of Du 2018 (34).

**
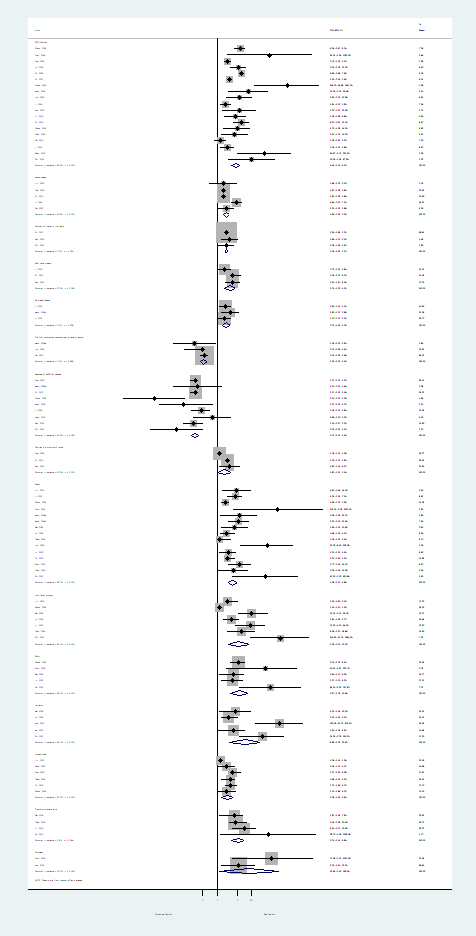
**

**Supplementary Figure S2.** Summary of all risk factors.
